# Identification and characterization of microRNAs in *Phaseolus vulgaris *by high-throughput sequencing

**DOI:** 10.1186/1471-2164-13-83

**Published:** 2012-03-06

**Authors:** Pablo Peláez, Minerva S Trejo, Luis P Iñiguez, Georgina Estrada-Navarrete, Alejandra A Covarrubias, José L Reyes, Federico Sanchez

**Affiliations:** 1 Departamento de Biología Molecular de Plantas, Instituto de Biotecnología, Universidad Nacional Autónoma de México, Cuernavaca, Morelos, Mexico

## Abstract

**Background:**

MicroRNAs (miRNAs) are endogenously encoded small RNAs that post-transcriptionally regulate gene expression. MiRNAs play essential roles in almost all plant biological processes. Currently, few miRNAs have been identified in the model food legume *Phaseolus vulgaris *(common bean). Recent advances in next generation sequencing technologies have allowed the identification of conserved and novel miRNAs in many plant species. Here, we used Illumina's sequencing by synthesis (SBS) technology to identify and characterize the miRNA population of *Phaseolus vulgaris*.

**Results:**

Small RNA libraries were generated from roots, flowers, leaves, and seedlings of *P. vulgaris*. Based on similarity to previously reported plant miRNAs,114 miRNAs belonging to 33 conserved miRNA families were identified. Stem-loop precursors and target gene sequences for several conserved common bean miRNAs were determined from publicly available databases. Less conserved miRNA families and species-specific common bean miRNA isoforms were also characterized. Moreover, novel miRNAs based on the small RNAs were found and their potential precursors were predicted. In addition, new target candidates for novel and conserved miRNAs were proposed. Finally, we studied organ-specific miRNA family expression levels through miRNA read frequencies.

**Conclusions:**

This work represents the first massive-scale RNA sequencing study performed in *Phaseolus vulgaris *to identify and characterize its miRNA population. It significantly increases the number of miRNAs, precursors, and targets identified in this agronomically important species. The miRNA expression analysis provides a foundation for understanding common bean miRNA organ-specific expression patterns. The present study offers an expanded picture of *P. vulgaris *miRNAs in relation to those of other legumes.

## Background

Small non-coding RNAs (sncRNAs) have been recognized as an important class of gene expression regulators. MicroRNAs (miRNAs) are ~21 nucleotide (nt) sncRNAs that regulate a multitude of biological processes in plants, including development, metabolism, stress responses, defense against pathogens, and maintenance of genome integrity [1]. MiRNAs direct cleavage or translational inhibition of a specific messenger RNA (mRNA) based on base-pairing complementarity between the miRNA and the target mRNA. MiRNAs are derived from imperfectly matched stem-loop structures that are formed from single-stranded primary miRNA transcripts (pri-miRNAs). MiRNA genes are RNA polymerase II transcription units that give rise to pri-miRNAs [2]. Pri-miRNAs are preferentially processed in the nucleus by an RNaseIII-type enzyme DICER-LIKE1 (DCL1) to release the precursor miRNAs (pre-miRNAs) [3]. Most plant pre-miRNAs produce a single miRNA/miRNA* duplex; the exceptions are some miR159 and miR319 loci [4]. These small RNA duplexes are subsequently 2'-*O*-methylated by the nuclear HUA ENHANCER 1 (HEN1) protein, preventing miRNA turnover, and are exported to the cytoplasm by the plant homolog of exportin-5 HASTY (HST) [5-7]. Finally, one of the strands of each duplex is incorporated into the RNA-Induced Silencing Complex (RISC) to guide gene silencing [8]. RISCs contain a member of the ARGONAUTE (AGO) protein family to direct the endonucleolytic cleavage of target RNAs. AGO1 is the predominant carrier of plant miRNAs [9]. Thus, miRNAs are used by RISCs as templates for recognizing complementary mRNA to regulate gene expression.

Since the first plant miRNAs were reported in *Arabidopsis thaliana *in 2002, there has been an exponential growth of identified miRNAs in a diverse number of plant species [10]. The miRNA database miRBase (release 16, Sept 2010) contains 15,172 microRNA loci from 142 species. Plant miRNA loci belonging to 43 species correspond to 22.4% of the total mature miRNA entries. The plant species with the most miRNA loci identified are *Oryza sativa*, *Medicago truncatula*, *Arabidopsis thaliana*, *Physcomitrella patens*, *Glycine max*, *Sorghum bicolor*, *Populus trichocarpa*, and *Zea mays*. These 8 widely studied plant model species contribute 65.8% of the plant mature miRNA entries. On the other hand, 15 plant species, including *Phaseolus vulgaris*, have fewer than 10 reported miRNA loci in miRBase.

Identification of *P. vulgaris *microRNAs was first performed using an *in silico *approach [11]. The eight miRNA loci reported for *P. vulgaris *in miRBase were identified by Arenas-Huertero et al. (2009) in different organs and growth conditions, although the first mature miRNA sequence ever cloned and characterized for *P. vulgaris *was pvu-miR399a [12]. This group of eight miRNAs in miRBase comprises miR2118, miR159a, miR1514a, miR482, miR2119, miR166a, miR319c, and miR399a. Besides these miRNAs and their stem-loop precursors, another 19 mature miRNA sequences have been identified [13]. In addition, Valdés-López et al. (2010) analyzed the expression of 68 miRNAs under several abiotic stress conditions in leaves, roots and nodules of *P. vulgaris *based on a hybridization approach using miRNA macroarrays. The macroarrays contained probes for 9 miRNAs previously reported for common bean, 24 conserved miRNAs also identified in other legumes and 35 miRNAs found in soybean [11,13,14].

The first studies to identify plant miRNAs employed the traditional Sanger sequencing method. This was also the case for the study performed by Arenas-Huertero et al. (2009) to identify miRNA sequences in *Phaseolus vulgaris*. Despite the Sanger sequencing method's usefulness and importance in scientific research, it has several limitations with regard to miRNA identification [15,16]. For example, low-abundance miRNAs, such as many non-conserved miRNAs, are inefficiently detected by this small-scale sequencing method. Introduction of high-throughput next-generation sequencing (NGS) technologies has increased the number of miRNAs identified by allowing small RNA population analysis on a massive scale (RNA-seq). Deep-sequencing technologies have identified a larger number of novel miRNAs due to their ability to generate millions of reads with a determined length [17]. Moreover, the population of short sequence reads produced allows the identification of differential processing of stem-loop precursors by DICER-LIKE1, and mature miRNA isoforms within and between species. Likewise, relative abundance associated with small RNAs estimated by these technologies has permitted a higher level of confidence for miRNA annotation.

Legumes such as common bean are valuable crops for worldwide consumption because they are rich in protein and constitute a high calorie food source. Involvement of miRNAs in biological processes like nutrient balance, development, reproduction and plant-microbe interaction, makes its research crucial for improvement of staple crops. Up to now, identification and characterization of *Phaseolus vulgaris *miRNAs have been very limited. In this study, Illumina's sequencing by synthesis (SBS) technology was used to examine conserved, novel and species-specific common bean miRNAs comprehensively based on small RNA libraries generated from leaves, roots, seedlings and flowers. A detailed analysis of mature, mature-star (the complementary strand of the mature miRNA), isoforms and novel common bean miRNAs found in different organs is reported. Stem-loop precursors and target sequences for several common bean miRNAs were also determined from public databases. Overall, this work serves to extend our knowledge of the common bean miRNA population and to spotlight new miRNA variants found in different organs. It facilitates comparisons between common bean miRNAs and those found in other legumes and plants.

## Results

### Small RNA sequencing analysis

**Figure 1:**
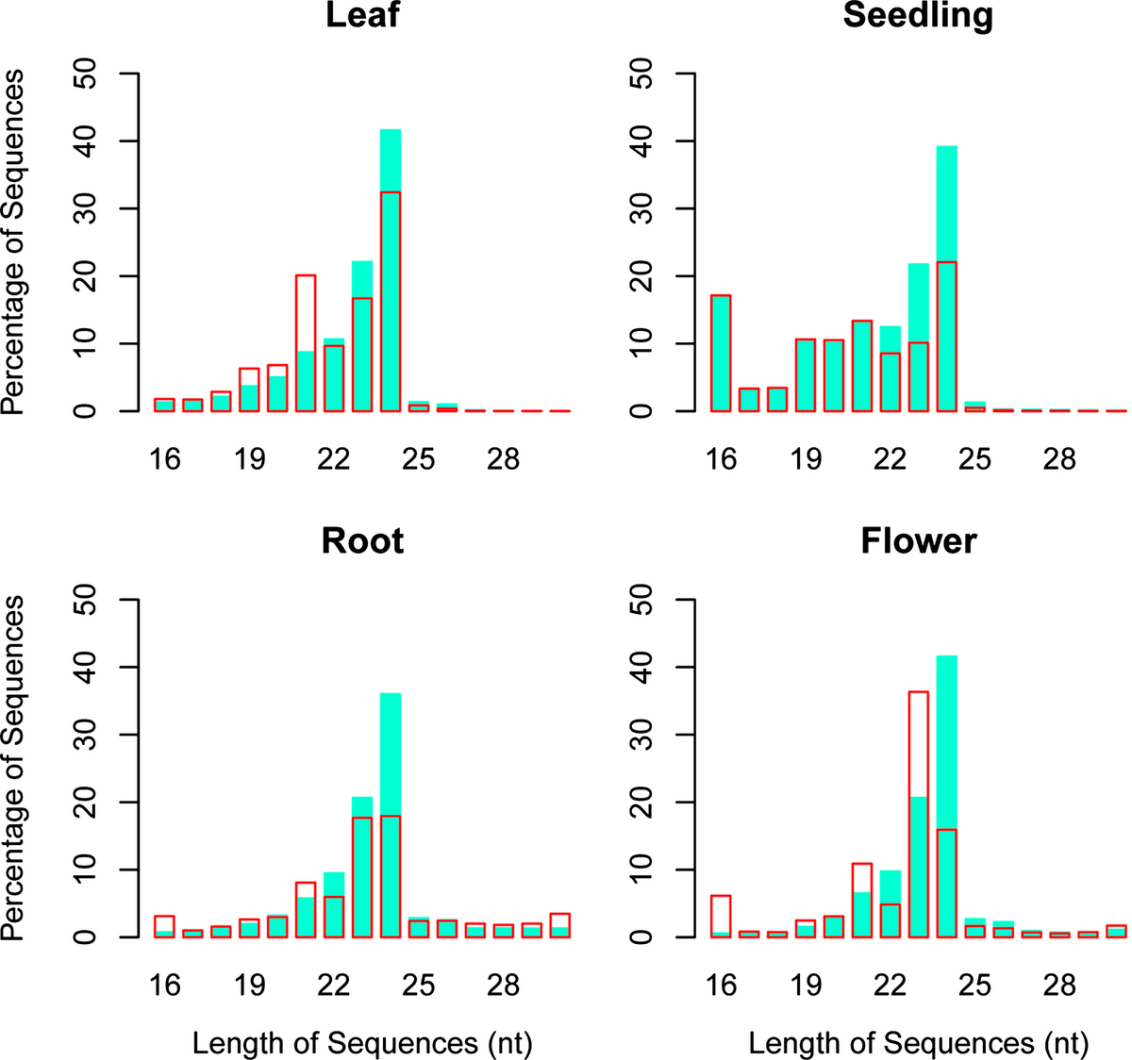
**Sequence length distribution of *P. vulgaris *sRNAs**. Size distribution in small RNA libraries. Average percentage (Y-axis) of unique (filled blue bars) and redundant (non-filled red bars) sequences of 16-30 nt length (X-axis) for each of the four sequenced libraries.

In order to identify novel and conserved miRNAs in common bean, we generated four small RNA libraries from leaves (LL), roots (RL), seedlings (SL) and flowers (FL) using the Genome Analyzer II and the Illumina Cluster Station (Illumina Inc, USA). Small RNA sequencing yielded more than eighty million raw sequence reads (Table 1). After removing low-quality sequences, adapters, and small sequences (< 16 nt long), 79.5% of the raw reads were left. These remaining sequences represent 3,372,753 (LL), 4,187,414 (RL), 4,015,702 (SL), and 3,453,543 (FL) unique sequence tags. The small RNA length distribution (16-30 nt) of each library showed that the most abundant and diverse species were those 21-24 nt in length, a typical size range for Dicer-derived products (Figure 1). The 24 nt class was the most diverse in all four libraries, followed by the 21 nt class, which was the second most abundant class in the LL library (20%), and the third most abundant class in the SL (13.4%), RL (8%), and FL (10.8%) libraries. Filtering sequences for removal of non-coding RNAs such as tRNA, rRNA, snoRNA or snRNA was performed using the RNA families database Rfam because of the limited availability of *P. vulgaris *genome sequences. After removing miRNA sequences from Rfam, the remainder of the database (Rfam 10.0) was used to eliminate fragments corresponding to non-coding RNAs. Alignment against the four small RNA libraries was carried out using BLASTN and only perfect matches were removed from the libraries (Table 1).

**Table 1:** Summary of small RNA sequencing data analysis

	Leaves	Roots	Seedlings	Flowers
Total raw reads	16869046	20464127	17188077	27937376

High-quality reads^a^	11797480	16722005	16846110	20238107

Unique sequence tags	3372753	4187414	4015702	3453543

Total Rfam matching sequences	757788	5653553	5307793	9726833

Unique Rfam matching sequences	51227	60121	224240	96726

Perfect miRNA matching sequences^b^	265161	307003	582159	1415225

Total unfiltered isoform sequences	798835	287027	467691	228556

Total miRNA isoform sequences	744348	200630	335274	60167

^a^Sequences after quality, adapter and size filters. ^b^Total sequences from conserved miRNAs.

### Identification of conserved miRNAs

To identify conserved miRNAs in the common bean small RNA libraries, unique mature plant miRNA sequences were extracted from miRBase (Release 16). BLASTN and SSAHA2 (Sequence Search and Alignment by Hashing Algorithm) tools were used separately for alignment of small RNA datasets against mature miRNA sequences in search of previously reported miRNAs with exactly the same size and nucleotide composition [18,19]. Both alignment tools found the same number of hits. MiRNAs that were detected in just one library or that totalled fewer than 15 absolute appearances in all libraries combined were removed (Additional file 1).

**Figure 2:**
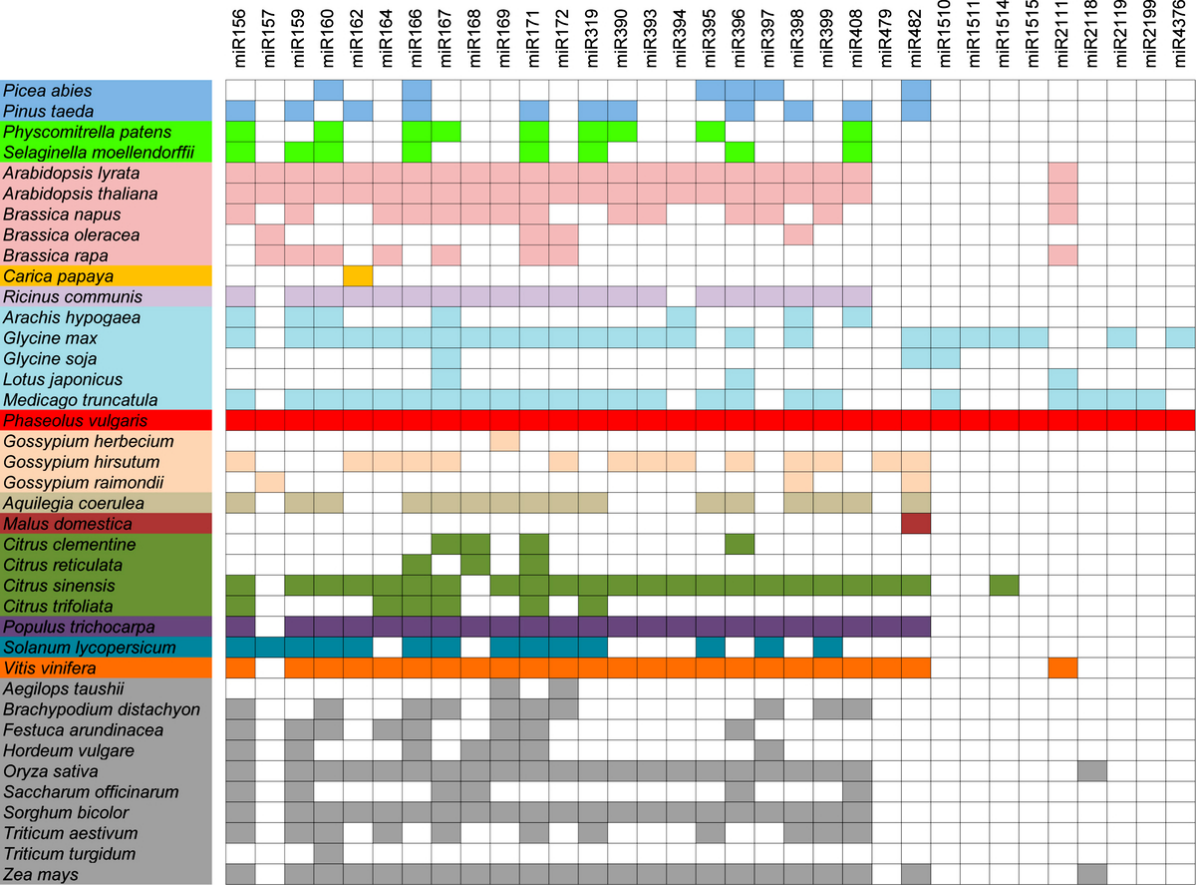
**Conserved miRNA families in *P. vulgaris *and across-species**. Evolutionary conservation of thirty-three miRNA families identified in *P. vulgaris *within plant species reported in miRBase (v.16). Closely related species are shown in the same color. Colored boxes denote the presence of the indicated miRNA family.

In total, 109 conserved miRNAs (Table 2) belonging to 29 families were identified in common bean. Fourteen of the conserved miRNAs were mature-star miRNAs (Table 3). Ten were detected in just two libraries, twenty five in three libraries, and seventy four in all four libraries. Twenty five of the families detected are highly conserved among plant species (Figure 2). Nine conserved families identified in common bean, including miR156, miR159, miR319, miR160, miR166, miR171, miR408, miR390 and miR395, were present in the common ancestor of all embryophytes [20]. Additionally, the miR396 family identified in all tracheophytes, and a pair of miRNA families detected in seed plants, miR397 and miR398, were present in *P. vulgaris*. Another nine identified are present in all angiosperm lineages: miR162, miR164, miR167, miR168, miR169, miR172, miR393, miR394, and miR399. The miR2111 family, which has been identified only in eudicot species, and the miR2118 family which has been found in monocots and in *Fabaceae*, were also identified in common bean. Finally, among the deeply conserved miRNA families detected, miR157, closely related to miR156, is present in *Brassicaceae*, *Solanaceae, Malvaceae *and *Fabaceae*.

**Table 2:** Conserved miRNAs from common bean

miRNA family	Reference miRNA	Sequence (5'- 3')	Length (nt)	Reads				
				
				LL	FL	RL	SL	Total
156	ahy-miR156a	UGACAGAAGAGAGAGAGCAC	20	56	15	8	41	120
	
	ahy-miR156c	UUGACAGAAGAGAGAGAGCAC	21	94	3512	3	317	3926
	
	ath-miR156a	UGACAGAAGAGAGUGAGCAC	20	82529	63	3705	14478	100775
	
	ath-miR156g	CGACAGAAGAGAGUGAGCAC	20	74	0	3	10	87
	
	bna-miR156a	UGACAGAAGAGAGUGAGCACA	21	112	21	2	26	161
	
	ptc-miR156k	UGACAGAAGAGAGGGAGCAC	20	47	0	6	2	55
	
	vvi-miR156e	UGACAGAGGAGAGUGAGCAC	20	30	0	1	1	32
	
	zma-miR156k	UGACAGAAGAGAGCGAGCAC	20	183	0	14	10	207

157	ath-miR157a	UUGACAGAAGAUAGAGAGCAC	21	3114	2787	4370	4839	15110
	
	ath-miR157d	UGACAGAAGAUAGAGAGCAC	20	361	30	26	89	506

159	aqc-miR159	UUUGGACUGAAGGGAGCUCUA	21	33	312	43	89	477
	
	pvu-miR159a.1	UUUGGAUUGAAGGGAGCUCUA	21	67007	1082688	180739	453692	1784126
	
	ath-miR159b	UUUGGAUUGAAGGGAGCUCUU	21	3	438	135	178	754
	
	gma-miR159c	AUUGGAGUGAAGGGAGCUCCG	21	0	0	1044	25	1069
	
	gma-miR159d	AGCUGCUUAGCUAUGGAUCCC	21	78	148	202	240	668
	
	osa-miR159a.1	UUUGGAUUGAAGGGAGCUCUG	21	37	224	31	148	440
	
	osa-miR159f	CUUGGAUUGAAGGGAGCUCUA	21	57	666	298	182	1203
	
	pta-miR159a	UUGGAUUGAAGGGAGCUCCA	20	0	3	0	25	28

160	ath-miR160a	UGCCUGGCUCCCUGUAUGCCA	21	136	5160	5474	104	10874
	
	bdi-miR160	UGCCUGGCUCCCUGUAUGCC	20	9	212	225	5	451
	
	osa-miR160f	UGCCUGGCUCCCUGAAUGCCA	21	9	3	240	2	254

162	ath-miR162a	UCGAUAAACCUCUGCAUCCAG	21	597	583	517	1192	2889
	
	zma-miR162	UCGAUAAACCUCUGCAUCCA	20	14	22	15	30	81

164	ath-miR164a	UGGAGAAGCAGGGCACGUGCA	21	7245	5621	3628	1229	17723
	
	ath-miR164c	UGGAGAAGCAGGGCACGUGCG	21	49	71	91	122	333

166	gma-miR166a	UCGGACCAGGCUUCAUUCCCC	21	5333	53276	20481	15163	94253
	
	ctr-miR166	UCGGACCAGGCUUCAUUCCCCC	22	2	18	10	10	40
	
	osa-miR166e	UCGAACCAGGCUUCAUUCCCC	21	0	17	8	4	29
	
	vvi-miR166a	UCGGACCAGGCUUCAUUCC	19	93	205	166	382	846
	
	zma-miR166h	UCGGACCAGGCUUCAUUCCC	20	140	769	352	630	1891

167	ath-miR167a	UGAAGCUGCCAGCAUGAUCUA	21	661	2883	3251	90	6885
	
	ath-miR167d	UGAAGCUGCCAGCAUGAUCUGG	22	22003	1443	5196	626	29268
	
	bna-miR167a	UGAAGCUGCCAGCAUGAUCUAA	22	2	0	17	0	19
	
	ccl-miR167a	UGAAGCUGCCAGCAUGAUCUGA	22	2267	8164	2727	276	13434
	
	osa-miR167d	UGAAGCUGCCAGCAUGAUCUG	21	15021	20039	6209	378	41647
	
	ptc-miR167f	UGAAGCUGCCAGCAUGAUCUU	21	36	252	146	5	439

168	ath-miR168a	UCGCUUGGUGCAGGUCGGGAA	21	382	991	605	43	2021

169	ath-miR169a	CAGCCAAGGAUGACUUGCCGA	21	0	11	0	44	55
	
	ath-miR169b	CAGCCAAGGAUGACUUGCCGG	21	1339	8784	154	6351	16628
	
	gma-miR169d	UGAGCCAAGGAUGACUUGCCGGU	23	0	4	11	0	15
	
	gma-miR169e	AGCCAAGGAUGACUUGCCGG	20	133	47	62	316	558
	
	mtr-miR169c	CAGCCAAGGGUGAUUUGCCGG	21	3916	166	78	2	4162
	
	mtr-miR169d	AAGCCAAGGAUGACUUGCCGG	21	126	863	389	4	1382
	
	osa-miR169e	UAGCCAAGGAUGACUUGCCGG	21	1	29	1	16	47

171	ath-miR171b	UUGAGCCGUGCCAAUAUCACG	21	119	790	0	37	946
	
	sly-miR171d	UUGAGCCGCGCCAAUAUCAC	20	2	1	67	7	77
	
	zma-miR171b	UUGAGCCGUGCCAAUAUCAC	20	28	40	13	2	83
	
	zma-miR171f	UUGAGCCGUGCCAAUAUCACA	21	62	245	139	68	514

172	ath-miR172a	AGAAUCUUGAUGAUGCUGCAU	21	43	38	24	1	106
	
	ptc-miR172g	GGAAUCUUGAUGAUGCUGCAG	21	353	18985	200	51	19589
	
	sbi-miR172b	GGAAUCUUGAUGAUGCUGCA	20	1	193	7	0	201
	
	zma-miR172a	AGAAUCUUGAUGAUGCUGCA	20	344	565	622	36	1567

319	ath-miR319a	UUGGACUGAAGGGAGCUCCCU	21	0	1672	192	320	2184
	
	mtr-miR319	UUGGACUGAAGGGAGCUCCC	20	21	66392	4936	40730	112079
	
	osa-miR319a	UUGGACUGAAGGGUGCUCCC	20	0	19	5	3	27
	
	ppt-miR319a	CUUGGACUGAAGGGAGCUCC	20	1	1622	14	106	1743
	
	ppt-miR319c	CUUGGACUGAAGGGAGCUCCC	21	16	11934	643	3082	15675
	
	pta-miR319	UUGGACUGAAGGGAGCUCC	19	0	1917	201	1139	3257
	
	ptc-miR319e	UUGGACUGAAGGGAGCUCCU	20	0	22	58	45	125
	
	vvi-miR319e	UUUGGACUGAAGGGAGCUCCU	21	0	55	2	1	58
	
	vvi-miR319g	UUGGACUGAAGGGAGCUCCCA	21	0	285	23	53	361

390	ath-miR390a	AAGCUCAGGAGGGAUAGCGCC	21	299	1264	153	63	1779
	
	gma-miR390b	AAGCUCAGGAGGGAUAGCACC	21	46	25	193	124	388

393	ath-miR393a	UCCAAAGGGAUCGCAUUGAUCC	22	51	295	441	9	796
	
	osa-miR393	UCCAAAGGGAUCGCAUUGAUC	21	13	65	101	0	179

394	ath-miR394a	UUGGCAUUCUGUCCACCUCC	20	71	7473	339	482	8365
	
	vvi-miR394a	UUGGCAUUCUGUCCACCUCCAU	22	0	112	7	0	119

395	tae-miR395b	UGAAGUGUUUGGGGGAACUC	20	5	25	4	8	42

396	ath-miR396a	UUCCACAGCUUUCUUGAACUG	21	760	44655	10489	601	56505
	
	ath-miR396b	UUCCACAGCUUUCUUGAACUU	21	1	185	33	16	235
	
	gma-miR396d	AAGAAAGCUGUGGGAGAAUAUGGC	24	0	61	22	77	160
	
	gma-miR396e	UUCCACAGCUUUCUUGAACUGU	22	0	29	6	0	35
	
	ptc-miR396f	UUCCACGGCUUUCUUGAACUG	21	0	11	4	1	16
	
	vvi-miR396a	UUCCACAGCUUUCUUGAACUA	21	0	23	2	1	26
	
	vvi-miR396b	UUCCACAGCUUUCUUGAACU	20	742	19924	2108	823	23597

397	ath-miR397a	UCAUUGAGUGCAGCGUUGAUG	21	2057	13	5276	70	7416
	
	zma-miR397a	UCAUUGAGCGCAGCGUUGAUG	21	5	0	23	0	28

398	ahy-miR398	UGUGUUCUCAGGUCACCCCU	20	0	25	350	0	375
	
	osa-miR398b	UGUGUUCUCAGGUCGCCCCUG	21	3760	57	1717	7	5541

399	ath-miR399a	UGCCAAAGGAGAUUUGCCCUG	21	15	12	4	0	31
	
	ath-miR399b	UGCCAAAGGAGAGUUGCCCUG	21	35	121	22	2	180
	
	osa-miR399e	UGCCAAAGGAGAUUUGCCCAG	21	49	45	3	1	98

408	ath-miR408	AUGCACUGCCUCUUCCCUGGC	21	2083	3718	9050	167	15018
	
	bdi-miR408	AUGCACUGCCUCUUCCCUGG	20	5	25	67	0	97
	
	osa-miR408	CUGCACUGCCUCUUCCCUGGC	21	3	5	17	0	25
	
	ppt-miR408b	UGCACUGCCUCUUCCCUGGCU	21	0	5	55	0	60

482	gso-miR482a	UCUUCCCUACACCUCCCAUAC	21	7	318	85	2	412
	
	pvu-miR482	UCUUCCCAAUUCCGCCCAUUCC	22	5	291	54	0	350

1511	gma-miR1511	AACCAGGCUCUGAUACCAUG	20	978	625	449	1529	3581

1514	pvu-miR1514a	UUCAUUUUGAAAAUAGGCAUUG	22	188	5481	1282	311	7262

1515	gma-miR1515	UCAUUUUGCGUGCAAUGAUCUG	22	27	853	341	86	1307

2111	ath-miR2111a	UAAUCUGCAUCCUGAGGUUUA	21	48	211	255	0	514

2118	mtr-miR2118	UUACCGAUUCCACCCAUUCCUA	22	8	7	1	1	17
	
	pvu-miR2118	UUGCCGAUUCCACCCAUUCCUA	22	238	17300	13707	1771	33016

2119	gma-miR2119	UCAAAGGGAGUUGUAGGGGAA	21	85	42	1448	6	1581

Conserved miRNAs in common bean identified in leaves (LL), roots (RL), seedlings (SL) and flowers (FL).

**Table 3:** Conserved mature-star miRNAs from common bean

miRNA family	Reference miRNA	Sequence (5'- 3')	Length (nt)	Reads				
	
				LL	FL	RL	SL	Total
166	aly-miR166b*	GGACUGUUGUCUGGCUCGAGG	21	233	0	5	3	241
	
	aly-miR166g*	GGAAUGUUGUUUGGCUCGAGG	21	38	143	186	257	624
	
	zma-miR166a*	GGAAUGUUGUCUGGCUCGGGG	21	26	3	11	7	47
	
	gma-miR166a-5p	GGAAUGUUGUCUGGCUCGAGG	21	36866	3839	6253	23902	70860
	
	zma-miR166m*	GGAAUGUUGGCUGGCUCGAGG	21	55	655	50	3363	4123

167	ahy-miR167-3p	AGAUCAUGUGGCAGUUUCACC	21	155	16	84	11	266

168	aly-miR168a*	CCCGCCUUGCAUCAACUGAAU	21	80	22	8	3	113

171	aly-MIR171a*	UAUUGGCCUGGUUCACUCAGA	21	179	710	1	543	1433

172	aly-miR172c*	GGAGCAUCAUCAAGAUUCACA	21	10	352	19	1	382

390	aly-miR390a*	CGCUAUCCAUCCUGAGUUUCA	21	149	524	69	10	752
	
	gma-miR390a-3p	CGCUAUCCAUCCUGAGUUUC	20	14	3	0	2	19

396	aly-miR396a*	GUUCAAUAAAGCUGUGGGAAG	21	267	306	3931	791	5295

482	gma-miR482a-5p	AGAAUUUGUGGGAAUGGGCUGA	22	0	17	1	1	19
	
	pvu-miR482*	GGAAUGGGCUGAUUGGGAAGCA	22	1185	6	440	10	1641

Conserved mature-star miRNAs in common bean identified in leaves (LL), roots (RL), seedlings (SL) and flowers (FL).

Four other, less conserved miRNA families that were identified have been reported mainly in legumes, specifically in *Glycine max*. This is the case for miR1511, which had been previously detected only in soybean, although recent *P. vulgaris *miRNA studies pointed out its presence in common bean [13,14,21,22]. Here, it was confirmed that soybean and common bean share exactly the same sequence for this miRNA and that it is one of the most abundant miRNAs in these legumes (Table 2). The miR1514 and miR2119 families have been reported exclusively in legumes such as *Glycine max*, *Medicago truncatula *and *Phaseolus vulgaris*. Surprisingly, miR1515 was reported only in two species: *G. max *and *Citrus Sinensis*.

The *P. vulgaris *miRNAs with the most reads in all four libraries (ath-miR159a, mtr-miR319, ath-miR156a, ath-miR166a, zma-miR166c*, ath-miR396a, osa-miR167d, pvu-miR2118 and ath-miR167d) generally corresponded to the most abundant miRNAs detected in legumes like *M. truncatula*, *G. max*, and in other plants like *Vitis vinifera *[23-25]. It is worth noting that among these ten most abundant miRNAs there is a mature-star miRNA gma-miR166a-5p that has also been highly detected by both deep sequencing and microarray expression analysis in the shoot apical meristem (SAM) of soybean [26]. Also, it has been demonstrated that gma-miR166a and its mature-star miRNA have different expression patterns in the SAM, suggesting that they may play different roles in regulating leaf development [26]. The high number of reads detected for other mature-star sequences found in this study may also imply particular regulatory roles of gene expression conditioned by these small RNAs since inhibitory activity of mature-star species has been reported in animals and plants (Table 3) [27,28].

The miRNA families comprising the most miRNAs in common bean are miR156, miR159, miR166, miR167, miR169, miR319 and miR396. These highly conserved families, which generally have the most abundant miRNAs, have variable numbers of loci among the model legumes *G. max *and *M. truncatula *(Table 4) (miRBase, release 16). According to mature miRNA sequence differences, the predicted numbers of loci for *P. vulgaris *miRNA families have a higher Pearson's correlation coefficient with the numbers of loci reported for soybean (0.78) than with those identified in *M. truncatula *(0.21).

**Table 4:** Predicted number of loci in common bean miRNA families

miRNA family	Number of loci			miRNA family	Number of loci		
			
	*Pvu*	*Gma*	*Mtr*		*Pvu*	*Gma*	*Mtr*
156	6	7	9	396	5	5	2

157	1	0	0	397	2	0	0

159	6	4	1	398	1	2	3

160	2	1	5	399	3	0	17

162	1	1	1	408	2	0	0

164	2	1	4	479	1	0	0

166	2	2	8	482	3	2	0

167	5	7	1	1510	1	2	2

168	1	1	1	1511	1	1	0

169	6	5	17	1514	1	2	0

171	3	3	7	1515	1	1	0

172	2	3	1	2111	1	0	19

319	4	3	2	2118	2	0	1

390	3	3	1	2119	1	1	1

393	1	3	2	2199	2	0	1

394	1	2	0	4376	1	1	0

395	1	0	18				

Abbreviations stand for: *Pvu*, *Phaseolus vulgaris*; *Gma*, *Glycine max*; *Mtr*, *Medicago truncatula*.

### Identification of miRNA isoforms

Small non-coding RNAs such as microRNAs were initially thought to have a specific sequence of a defined length. Identification of more miRNAs from different species has revealed that there is variation in pre-miRNA processing. The miRBase (release16) dataset for plant miRNAs consists of small sequences of 17 (.08%), 18 (.13%), 19 (.88%), 20 (9.96%), 21 (67.06%), 22 (13.85%), 23 (1.10%), and 24 (6.91%) nucleotides in length. Ebhardt *et al. *(2010) demonstrated that a fifth of the annotated *Arabidopsis thaliana *miRNAs (miRBase, release 14) have a stable miRNA isoform of one or two nucleotides longer [29]. Also, several studies have revealed that the biological function of miRNA isoforms may differ from the function of their previously reported miRNAs due to differential associations with AGO proteins [30,31]. The legumes *M. truncatula *and *G. max*, which account for the majority of entries for the *Fabaceae *family in miRBase, present mature miRNA sequences ranging from 20-22 and 19-24 nucleotides in length, respectively.

With the aim of identifying *P. vulgaris *miRNA length isoforms and species-specific miRNA variants, all small RNA sequences remaining from previous analyses were aligned against miRBase, allowing at most two mismatches and/or two nucleotides in length difference. The total number of variants found for each library (Table 1) was subjected to a filter that consisted of choosing variants that had a total number of reads 50% greater than the number of total reads of their reference miRNA previously reported, so that low-abundance and probable non-functional variants were discarded. MiRNA isoforms were classified as length variants, non-conserved miRNA variants, or conserved miRNA variants (Table 5). Seven variants based only on length were detected. In all four *P. vulgaris *libraries, the 21-nt variant of gma-miR1511 is clearly predominant. In the case of the variant for gso-miR482a, it was highly expressed in the flower (FL) and root (RL) libraries. Unexpectedly, a variant for miRNA mtr-miR171c, a miRNA that was absent in all libraries, was detected mainly in flower. The high abundance of pvu-miR482* in common bean compared with other plant species was previously corroborated by northern blot [13]. As Table 5 shows, the high number of reads detected for this mature-star miRNA in all organs was produced by the 21-nt variant and not by the one reported in miRBase. As mentioned before, some miR159 and miR319 loci produce two miRNA/miRNA* duplexes. This was the case for the *P. vulgaris *miR159a stem-loop. The variant for pvu-miR159a.2 that is found closer to the loop is mainly a 19 nt miRNA, and its number of reads does not equate to the abundance of pvu-miR159a.1. The length variant gma-miR4376 increases the number of *P. vulgaris *miRNA families identified in this study because of its high number of reads. The miR4376 family had been identified only in soybean (Figure 2), and was registered in miRBase as a 22 nt miRNA [21]. The group of non-conserved miRNA variants contains nine variants from eight non-conserved miRNA families (Table 5). Considering the number of reads and that mature-miRNA homologs may present one or two mismatches, another three common bean miRNA families were identified: miR1510, miR479, and miR2199. For the miR1510 family, two variants were identified that can be produced from the same locus. This family has been reported only in soybean and *M. truncatula*, and was proposed to be conserved in common bean due to a 20 nt isoform [13,14,23]. The variant of csi-miR479, a miRNA detected in *Citrus sinensis*, *Vitis vinifera*, *Populus trichocarpa *and *Gossypium hirsutum *was particularly abundant in roots [25,32-34]. Interestingly, in this study two variants with the same length were detected for mtr-miR2199, one that was detected in all four libraries (A17 > G) and the other (C12 > T) expressed mostly in roots. A previous study in *M. truncatula *where this family was detected, reported the mature miRNA as the variant A17 > G and the genome derived-hairpin as A17, which accounts for its annotation in miRBase with an adenine in position 17 [35]. The miRNA sequence conserved in *Lotus japonicus *was the variant A17 > G also found in *P. vulgaris *[35]. The ten miRNA variants classified in the conserved miRNA variants group constitute candidates for new miRNAs of previously identified miRNA families (Table 5). The miRNA candidates with the most reads were the variants of osa-miR156l, csi-miR393, sly-miR171c, and mtr-miR169p.

**Table 5:** MiRNA isoforms from *P.vulgaris*

Variant group	Reference miRNA	Sequence (5'- 3')	Length (nt)	Reads				
	
				LL	FL	RL	SL	Total
Length variants	gma-miR1511	AACCAGGCTCTGATACCATG	20	978	625	449	1529	3581
	
	pvu-isomiR 1511	AACCAGGCTCTGATACCATGA	21	14902	13725	7707	17239	53573
	
	gso-miR482a	TCTTCCCTACACCTCCCATAC	21	7	318	85	2	412
	
	pvu-isomiR 482a	TCTTCCCTACACCTCCCATACC	22	369	17616	5564	305	23854
	
	mtr-miR171c	TGATTGAGCCGTGCCAATATT	21	0	0	0	0	0
	
	pvu-isomiR 171a	TGATTGAGCCGTGCCAATA	19	115	1390	60	57	1622
	
	pvu-miR482*	GGAATGGGCTGATTGGGAAGCA	22	1185	6	440	10	1641
	
	pvu-isomiR 482*	GGAATGGGCTGATTGGGAAGC	21	714044	3806	173153	1	1207244
	
	pvu-miR159a.2	CTTCCATATCTGGGGAGCTTC	21	1	0	0	0	1
	
	pvu-isomiR 159a	CTTCCATATCTGGGGAGCT	19	13	263	157	23	456
	
	gma-miR4376	TACGCAGGAGAGATGACGCTGT	22	1	1	0	0	2
	
	pvu-isomiR 4376	TACGCAGGAGAGATGACGCTG	21	3841	1090	0	398	5329
	
	mtr-miR171b	TGATTGAGCCGCGTCAATATC	21	0	0	0	0	0
	
	pvu-isomiR171b	TCTGATTGAGCCGCGTCAATA	21	101	1	79	10	191

Non conserved variants	ath-miR858	TTTCGTTGTCTGTTCGACCTT	21	0	0	0	0	0
	
	pvu-isomiR 858	CTCGTTGTCTGTTCGACCTTG	21	37	351	0	26	414
	
	csi-miR479	TGTGATATTGGTTCGGCTCATC	22	0	0	0	0	0
	
	pvu-isomiR479	TGTGATATTGGTTTGGCTCA	20	58	1	1588	11	1658
	
	gma-miR1510a-3p	TTGTTGTTTTACCTATTCCACC	22	0	0	0	0	0
	
	pvu-isomiR1510a	TGTTGTTTTTCCTATTCCACC	21	463	877	902	28	2270
	
	gma-miR1510b	TGTTGTTTTACCTATTCCACC	21	0	0	4	0	4
	
	pvu-isomiR1510b	TTGTTTTTCCTATTCCACCAA	21	3313	17893	8413	222	29841
	
	mtr-miR2199	TGATACACTAGCACGGATCAC	21	8	0	0	0	8
	
	pvu-isomiR 2199a	TGATACACTAGCACGGGTCAC	21	16	1323	799	59	2197
	
	pvu-isomiR 2199b	TGATACACTAGTACGGATCAC	21	2586	0	0	5	2591
	
	mtr-miR2597	TTTGGTACTTCGTCGATTTGA	21	0	0	0	0	0
	
	pvu-isomiR2597	TTTGGTACTTCCTTGATTTGA	21	0	22	253	0	275
	
	ppt-miR894	CGTTTCACGTCGGGTTCACC	20	1	3	1	0	5
	
	pvu-isomiR894	CGTTTCACGTCAGGTTCACCA	21	15	6	0	0	21
	
	pta-miR1310	GGCATCGGGGGCGTAACGCCCCT	23	0	0	0	0	0
	
	pvu-isomiR1310	GGCATCGGGGGCGCAACGCCC	21	33	5	0	0	38

Conserved variants	ctr-miR166a	TCGGACCAGGCTTCATTCCCCC	22	2	18	10	10	40
	
	pvu-isomiR166a	TCGGACCAGGCTTCCTTCCCC	21	113	371	241	63	788
	
	osa-miR156l	CGACAGAAGAGAGTGAGCATA	21	0	0	0	0	0
	
	pvu-isomiR 156a	TGACAGAAGAGAGTGAGCA	19	2682	7	334	222	3245
	
	mtr-miR164d	TGGAGAAGCAGGGCACATGCT	21	0	0	0	0	0
	
	pvu-isomiR164a	TGGAGAAGCAGGACACATGC	20	58	64	535	12	669
	
	vvi-miR156e	TGACAGAGGAGAGTGAGCAC	20	30	0	1	1	32
	
	pvu-isomiR 156b	TGACAGACGAGAGTGAGCAC	20	261	0	2	2	265
	
	csi-miR393	ATCCAAAGGGATCGCATTGATC	22	1	0	0	0	1
	
	pvu-isomiR393	TTCCAAAGGGATCGCATTGA	20	911	581	667	4	2163
	
	ctr-miR171	TTGAGCCGCGTCAATATCTCC	21	0	0	0	0	0
	
	pvu-isomiR171c	TTGAGCCGCGTCAATATCTCA	21	30	151	129	58	368
	
	mtr-miR169p	TGAGCCAGGATGGCTTGCCGG	21	0	0	0	0	0
	
	pvu-isomiR169a	TGAGCCGGGATGGCTTGCCGG	21	2	228	26	97	353
	
	ptc-miR396f	TTCCACGGCTTTCTTGAACTG	21	0	11	4	1	16
	
	pvu-isomiR396a	TTCCACCGCTTTCTTGAACTG	21	11	10	3	0	24
	
	zma-miR398a	TGTGTTCTCAGGTCGCCCCCG	21	2	0	2	0	4
	
	pvu-isomiR398a	TGTGTTCTCAGGCCGCCCCTG	21	21	0	18	0	39
	
	sly-miR171c	TATTGGTGCGGTTCAATGAGA	21	0	0	0	0	0
	
	pvu-isomiR171d	TATTGGTCCGGTTCAATGAGA	21	353	386	0	192	931

*Phaseolus vulgaris *miRNA isoforms were classified as length variants, non-conserved miRNA variants, or conserved miRNA variants. Variants were named with the prefix pvu-isomiR followed by the miRNA family number of the reference miRNA. Underlined bases denote differences in length and/or composition of bases between variants and reference miRNAs.

### Identification of stem-loop precursors

Plants have a more diverse population of sncRNAs than do animals mainly because of particular RNA polymerases. A criterion that supports miRNA annotation is the identification of a stem-loop precursor from which the duplex of miRNA/miRNA* is excised. A search for potential miRNA precursors involves secondary structure prediction analysis of genomic DNA or EST (Expressed Sequence Tag) sequences that match perfectly with determined miRNAs. Although plant miRNA stem-loops are more variable in length and structure than are animal pre-miRNAs, several characteristics are conserved among plant precursors. To identify common bean stem-loop precursors, miRNAs from Table 2, Table 3, Additional file 1, and Table 5 were aligned against all *P. vulgaris *ESTs and GSSs (Genomic Survey Sequences) from NCBI in search of perfect alignments. EST and GSS candidates were subjected to secondary structure prediction analysis using the *mfold *program with its default parameters [36]. Only the lowest energy structures were selected as described by Reinhart et al. (2002).

**Figure 3:**
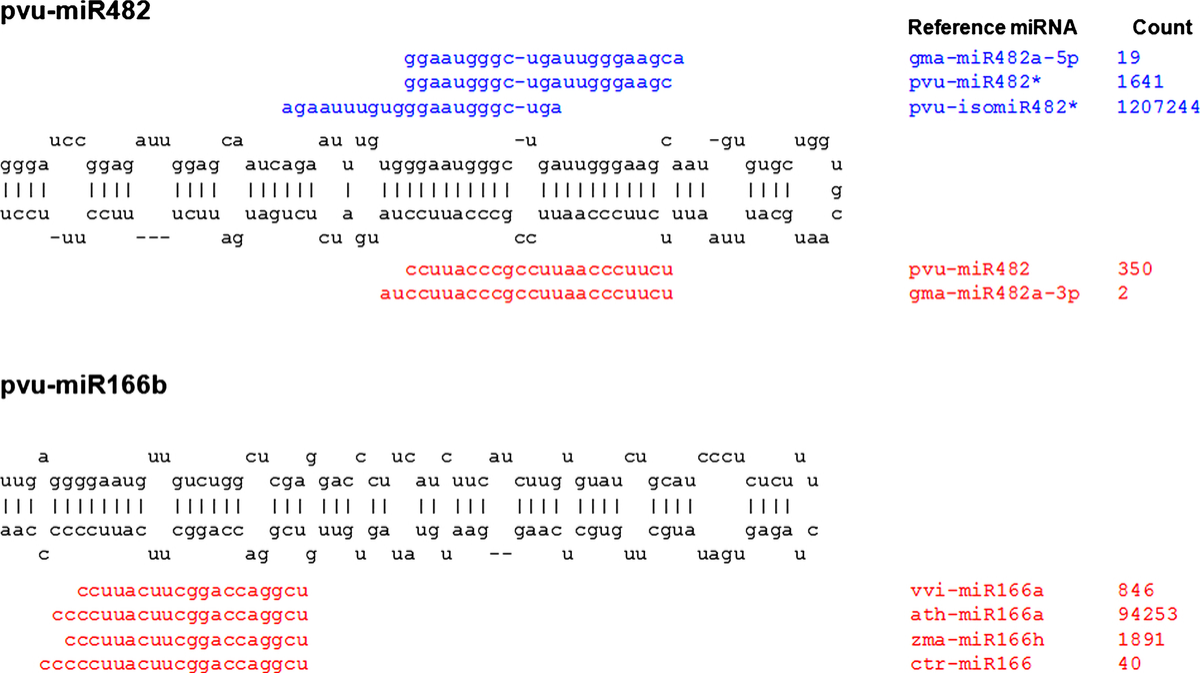
**Differential processing of *P. vulgaris *pre-miRNAs**. Stem-loop precursors of pvu-miR482 (acc.: MI0010702) and novel pvu-miR166b (gi: 171606821) pre-miRNAs were aligned against possible derived mature (red) and mature-star (blue) miRNA sequences. Count data number represents the total number of reads found in all four libraries (right column).

Secondary structure prediction analysis of *P. vulgaris *sequences resulted in the identification of eleven new stem-loop precursors belonging to eight conserved miRNA families (Figure 3 for an example and Additional file 2 for complete set). The precursor for the miR171 family was the precursor for the variant of ctr-miR171 (pvu-isomiR171c). The other miRNA families with a precursor identified were: miR166, miR167, miR156, miR157, miR398, miR408, and miR168. Taking into account previously reported *P. vulgaris *precursors, a total of 39 miRNAs can be predicted by the processing of common bean precursors, including pvu-isomiR171c (Additional file 2) [13]. The miRNAs ath-miR319c, osa-miR156k, gma-miR156f, ath-miR398a, and gma-miR482a-3p, discarded because they appeared in just one library or gave rise to fewer than 15 reads in all libraries, aligned perfectly with their respective precursors. Pvu-miR482*, aly-miR168a* and gma-miR166a-5p were also found in the precursor sequences. Therefore, common bean isoforms pvu-isomiR159a and pvu-isomiR482* were most likely derived also from these precursors.

### Identification of novel microRNAs

From previous analyses, *P. vulgaris *miRNAs identical to those already present in miRBase or close homologs were identified. Numerous studies have described novel miRNAs in several plant species based on small RNA high-throughput sequencing results. However, this analysis is usually performed based on a reference genome that allows for mapping of a given small RNA to a genomic location and retrieval of adjoining sequence to help with secondary structure prediction of a miRNA precursor. In the present study, the sequencing reads remaining after removing known miRNAs were employed to scan the collection of ESTs and genomic sequences available from NCBI and PlantGDB in search of potential miRNAs using the miRDeep software [37]. This program searches for those small RNAs present in the sequencing reads that can be mapped to a given reference sequence. Selected sequences are searched for their potential RNA secondary structure to identify those that can be folded as hairpin structures typical of plant miRNA precursors. Additionally, the mapped small RNAs should be located in the stem region of the folded RNA, and a higher probability score is awarded to a given candidate if there are sequencing reads corresponding to the predicted miRNA* region.

**Figure 4:**
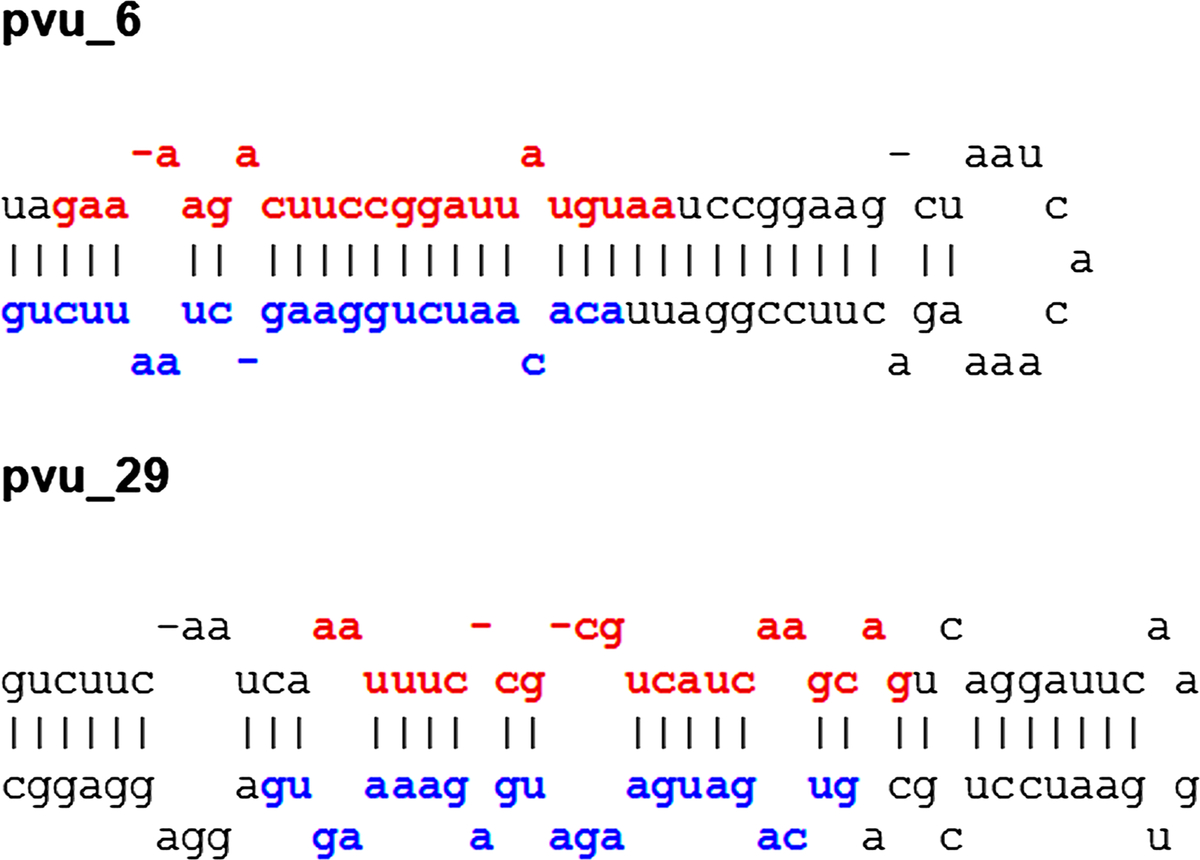
**Predicted stem-loop precursors of two novel miRNAs**. Secondary structure prediction for candidate novel miRNAs pvu_6 and pvu_29 were carried out with *mfold*. Mature (red) and mature-star (blue) miRNAs correspond to predicted mature and mature star miRNA sequences from miRDeep.

The analysis for identification of novel miRNAs was carried out with 95030, 74759, 87956 and 65741 unique sequences from the leaf, root, seedling and flower small RNA libraries, respectively, and a total of 124894 ESTs and genomic sequences. A total of 29 candidates for new miRNA precursors were identified (Figure 4 for examples and Additional file 3 for complete set of candidate miRNAs). Four of the candidate precursors included reads for a mature miRNA and a putative miRNA* and the other 25 had only reads consistent with the predicted mature miRNA. The small number of ESTs and amount of genomic data available for *P. vulgaris *limits the number of candidates recovered by this analysis. The forthcoming availability of a complete genome should reveal a more comprehensive picture of the miRNA genes present in the common bean genome.

### Identification of miRNA targets in the genus *Phaseolus*

Plant miRNAs typically have higher sequence complementarity with their target mRNAs compared with miRNA-target interactions in animals [38]. MiRNA target prediction in plants is based on this high degree of complementarity between miRNAs and targets [39]. Many plant miRNA:mRNA duplexes exhibit paired nucleotides in the microRNA 5' region. However, some conserved plant miRNA-target interactions with unpaired nucleotides in the 'seed' region have been identified, for instance miR398a-CSD2 and miR396a-GRL7/GRL8/GRL9 in *A. thaliana *[39]. In this study, the RNAhybrid program as described by Alves et al. (2009) for prediction of *Phaseolus *miRNA targets was used (See methods) [40]. RNAhybrid predicts the energetically most favorable miRNA:mRNA hybrids according to user preferences [41]. All *Phaseolus *ESTs (NCBI) were evaluated as targets of miRNAs from Table 2, Table 3, and Table 5. Identification analysis for *P. vulgaris *miR390 non-coding transcript target TAS3 was done separately (See methods) [42]. Once miRNA:mRNA hybrids were obtained, ESTs were aligned against plant sequences of the UniProt Knowledgebase (UniProtKB) (release 2011_01) using BLASTX for annotation.

Target prediction analysis identified 194 ESTs annotated as established target gene families in plants (Additional file 4). Thirty seven ESTs were previously reported by Arenas-Huertero et al. (2009) and most are ESTs with small minimum free energies (MFEs) (data not shown). The conserved miRNA families for which conserved targets were found are: miR156/miR157, miR160, miR164, miR167, miR168, miR169, miR171, miR172, miR319, miR393, miR395, miR397, miR398 and miR408. The number of ESTs found for each gene family are: SBP(7), ARF(18), NAC(1), AGO1(1), NFY(14), SCL(2), AP2(8), TCP(6), TIR/F-box-AFB(5), ATP sulfurylases(14), Laccases(1), COX/SOD(66) and Plastocyanins(11). The miR156 and miR157 families share the same target EST sequences for Squamosa Binding Proteins (SBP). The MFEs for the conserved miRNA:mRNA hybrids range from -35.0 to -51.2 kcal/mol. Finally, among conserved miRNA targets, three ESTs homologous to AtTAS3, a target of miR390 family were identified (Additional file 4). In addition, 325 ESTs as putative targets for *P. vulgaris *conserved miRNAs, mature-star sequences and miRNA isoforms were identified (Additional file 5). Target prediction analysis for novel candidate miRNAs generated candidate targets for ten novel miRNAs represented by 177 ESTs (Additional file 6).

### Organ-specific expression of *P. vulgaris *miRNA families

Specific miRNA expression patterns are proposed to be a consequence of tissue-specific, cell-specific and/or stress-specific regulatory elements in promoters of plant microRNA (*MIRNA*) genes [43-45]. The expression of some conserved *MIRNA *genes and the relative abundance patterns of mature miRNAs in different organs or in different developmental stages are essential for proper cell differentiation and organ developmental regulation. Modification of *MIRNA *gene expression leads to severe developmental defects. Understanding expression patterns of microRNAs in plant organs is necessary to discern miRNA-mediated regulatory pathways. The frequency of miRNAs detected by high throughout sequencing methods serves for relative miRNA expression estimation [46]. Moldovan et al. (2009), comparing *Arabidopsis *root miRNA frequencies against leaf and whole plant reads represented in the Arabidopsis Small RNA Project database (ASRP), found that most miRNA families have organ-specific expression [47,48].

**Figure 5:**
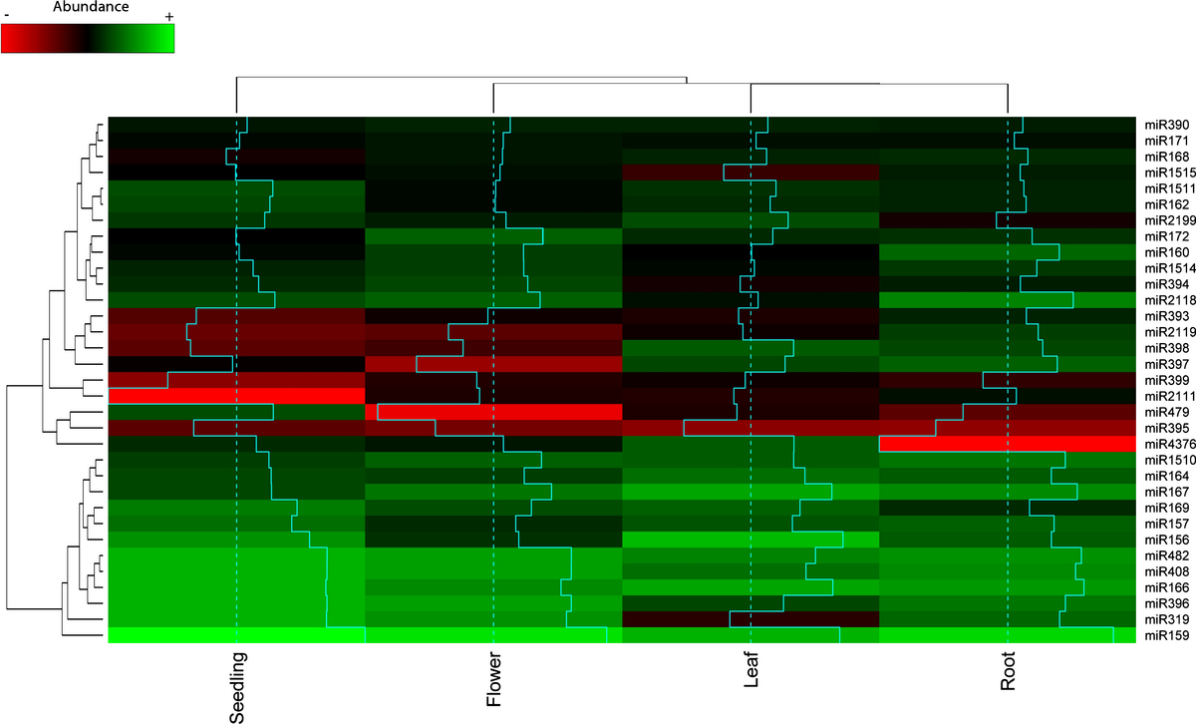
**Hierarchical cluster analysis (HCA) of *P. vulgaris *miRNA families**. Frequencies of conserved miRNA families were used to analyze relative miRNA family expression in leaf, seedling, root and flower. Hierarchical cluster analysis was performed on thirty-three conserved miRNA families (right). The color scale denotes abundant (green) and non-abundant (red) miRNA families according to transformed count data. Density plots are included in blue lines.

To explore organ-specific miRNA expression in *Phaseolus vulgaris*, the open-source R/Bioconductor software package DESeq was employed [49,50]. The DESeq package, supported by a model based on negative binomial distribution, estimates variance-mean dependence in count data from high-throughput sequencing assays and tests for differential expression. The miRanalyzer tool bases its differential expression module on the DESeq package and has been used to test for miRNA differential expression [51,52]. To explore organ-specific expression for miRNA families as a whole, frequencies for miRNAs of the same family were pooled together for each library and employed as input data. The five miRNAs that aligned perfectly with precursor sequences and were discarded because they appeared in just one library or totalled fewer than 15 reads in all libraries, as well as those newly identified from the isoform analysis, were also considered. Mature-star miRNAs were discarded. Count data was transformed with the DESeq package and hierarchical cluster analysis (HCA) was performed (Figure 5, see methods).

MiRNA families were clustered in two major groups: the group with fewer miRNAs consists of miRNA families that were abundantly expressed in all four libraries, and the other group contains miRNA families with differential expression patterns among organs. The accumulation patterns of miRNA families in leaf and root organs were more similar to each other than to the other two samples. The most abundant families of the analysis, especially in seedlings, were miR159, miR319, miR396, miR166, miR408, and miR482. The miR159 family has previously been reported as an abundant and widespread family in all plant organs. It is important to mention that the expression of miR319 was abundant in all organs except for leaf (Figure 5). Similar to miR319, in this study miR396 was less abundant in leaf than in the other three organs. Other quite abundant miRNA families were: miR1510, miR164, miR167, miR169, miR157 and miR156. Interestingly miR1510, a miRNA family detected only in legumes, was found in all four libraries.

In the group of families with a particular expression pattern, miR2119, miR2111, and miR479 were highly accumulated specifically in one organ. MiR2119 was detected especially in roots. This expression pattern correlates with previous northern blot analysis of roots, leaves, immature embryos and dry embryos from *P. vulgaris *[13]. Expression of miR2111 was observed mainly in roots and miR399 was only slightly expressed in leaves. Both miRNAs are induced under phosphorus (P) limiting conditions [12,53]. The miR479 family was preferentially expressed in seedlings. On the other hand, there were some interesting families that were poorly expressed in one particular organ. For example, miR168 was poorly expressed in seedlings, miR1515 in leaves, miR2199 in roots, miR394 in leaves, miR4375 in roots, and miR397 in flowers. Finally, HCA showed that miR395 was poorly expressed in all four small RNA libraries, consistent with its induction upon low sulphate conditions [54].

## Discussion

Identification of microRNAs in species with or without fully sequenced genomes has been revolutionized by high-throughput sequencing methods. High-throughput sequencing miRNA analysis in *Phaseolus vulgaris *will facilitate more particular and specific bean miRNA studies as well as legume sncRNAs research in this rapidly growing field. Although deep-sequencing experiments have become the major source supporting microRNA annotations, technical variations, transcript length bias and mapping bias have been reported with this approach for transcriptome analysis of coding RNAs (mRNAs) [17,55,56]. Studies to elucidate the number of miRNA molecules sequenced from a small RNA library are still needed for more accurate small RNA profiling studies. In terms of reads, the small RNA libraries sequenced here yielded a larger number of total raw reads to work with than did many other studies in which plant miRNAs have been identified; however, the total number of reads of identified miRNAs, including non-abundant and miRNA isoforms, constitutes only 0.047% of total raw reads. Further studies are needed to understand to what extent the 24-nt class representing siRNA populations, usually the most abundant and diverse class of sncRNAs sequenced in small RNA libraries, masks miRNA populations. Also, coverage analyses with fully sequenced genomes are needed to elucidate sequenced sample proportions of small non-coding RNAs such as tRNA, rRNA, snoRNA or snRNA.

Most miRNA families identified in this study in *P. vulgaris *are evolutionarily conserved in *Fabaceae*, particularly in the best-studied plants *M. truncatula *and *G. max*. MiRNA family miR157 has not been reported in these two legumes, probably because of the similarity of its sequence to that of miR156. Other miRNA families not reported in miRBase in *G. max *and *M. truncatula *are miR397 and miR408. The only *Fabaceae *where miR408 has been previously found was *Arachis hypogaea*. One miRNA of the bean miR397 family with the same sequence as ath-miR397 was detected herein with 7416 reads in total, so it is likely to be present in other legumes as well. MicroRNA miR2199 has been reported in miRBase only for *M. truncatula *and the variant found here for this miRNA in all four libraries of *P. vulgaris *was previously reported as miRS1 by Arenas-Huertero et al. (2009) because mtr-miR2199 was not yet reported. As well as in *Lotus japonicus*, the A17- > G (miRS1) variant was found in peanuts (*Arachis hypogaea L*.) [35,57].

It is interesting that miRNA families such as miR1511, miR1514 and miR1515 first detected in soybean, have not been found in other legumes besides *P. vulgaris*. In this regard, other miRNA families reported specifically in soybean (gma-miR1524, gma-miR1532, gma-miR1526, gma-miR1516 and gma-miR1513 and gma-miR1508) were detected by Valdés-López et al. (2010) using a hybridization approach (macroarrays) under several abiotic stress conditions in leaves, roots and nodules of *P. vulgaris*. These miRNAs from soybean were not detected by high-throughput sequencing, with the exception of two reads for gma-miR1508. In the Valdés-López et al. (2010) analysis, expression was detected for miRNAs miR1524, miR1526, miR1532 and miR1508 in common bean plants grown under sufficient nutrient conditions or stressed conditions. Family miRNAs gma-miR1513 and gma-miR1516 were detected only under stressed conditions. In addition, expression was detected in the macroarrays for an oligonucleotide probe designated as pvu-miR1509. This probe was based on a variant detected by Arenas-Huertero et al. (2009) that was not detected here because it has more than two mismatches with the reference miRNA gma-miR1509. The miR170 family, for which Valdés-López et al. (2010) detected expression in *P. vulgaris*, was neither found in this study. MiR170 is very similar to miR171, so it is possible that the macroarray probes hybridized with the same miRNA family.

MiRNA variants are considered to be a consequence of inaccuracies in Dicer pre-miRNA processing. Smaller variants with missing bases and low frequencies are viewed as degradation products or pyrophosphate sequencing errors. Herein, small RNA sequences from libraries were classified as miRNA isoforms only if they were similar to a reference miRNA reported in miRBase and had a significantly greater number of reads compared to those found for the reference miRNA in all four organs. From this isoform identification analysis, four more miRNA families (miR1510, miR2199, miR4376 and miR479) were added to the total number of conserved miRNA families identified in common bean. All of the variants found here for these four families were highly abundant in all four libraries. It is probable that miR1510 and miR2199 miRNA families are part of the *P. vulgaris *miRNA population, based on the two variants identified for each of these two families, along with their conservation in other species (see results) and previously experimental expression analyses [13,22].

Another family highly likely to be present in common bean is the miR4376 family. The variant found here for miR4376 was very abundant in common bean and has only one nucleotide missing relative to its reference miRNA found in soybean. On the other hand, further experimental and genomic sequences analyses are still needed to validate the variant identified in common bean for the miR479 family, which has two nucleotides missing and one mismatch compared with its reference miRNA csi-miRNA479. That is also the case for other less conserved miRNA families identified based on the isoform analysis, like miR858, miR2597, miR894 and miR1310. Most of the reference miRNAs for these less conserved miRNA family variants were poorly or not detected in the libraries.

In plant and animal microRNAs, 3' heterogeneity is the most frequent length variation. Most of the variants identified from the length variant group exhibit 3' heterogeneity. Sequence length heterogeneity for plant microRNAs has been proven to be essential for correct plant development and environmental responses. It is known that miR168 in *Arabidopsis thaliana *is produced in length variants of 21 and 22 nucleotides [31]. A decrease in abundance of the 21 nt variant reduces miR168 homeostasis and leads to developmental defects. The large number of reads detected for some variants in *P. vulgaris *suggests significant regulatory roles like detected for miR168 in *A. thaliana*. Using the pre-miRNA of pvu-miR482 (Figure 4) as an example, three variants generated for "mature-star" miRNAs (5p) and two variants generated for the mature miRNAs (3p) were observed. Considering the number of reads found for these variants, mature-star sequence variants were by far greater in abundance than their corresponding mature sequence variants. In particularl two mature-star sequence length variants, one previously reported in miRBase as pvu-miR482* of 22 nucleotides and the other here denoted as pvu-isomiR482* of 21 nucleotides, had 1641 and 1207244 total reads respectively. It is important to take into account that sequences for reference miRNA gso-miR482a and its highly abundant variant pvu-isomiR482a were detected, and that these variants necessarily have to be excised from another stem-loop precursor not yet identified. For this reason, it is possible that the variants pvu-miR482* and pvu-isomiR482* were generated from different loci, as actually happens for the two miR168 variants in *A. thaliana*. In addition to miRNAs previously reported in other plants and those present in closely related species such as soybean or *Medicago*, common bean may encode species-specific miRNAs. To address this question, sequencing reads were explored using the miRDeep algorithm [37]. Those candidates were favoured where in addition to a mature miRNA present in the stem region of the hairpin precursor, there was also evidence of a miRNA* sequence recovered. Next, candidate miRNAs having a plausible stem-loop precursor but without any miRNA* sequences in the libraries were obtained. It will be interesting to see to what extent these candidates can be confirmed as genuine miRNAs and whether they are involved in biological processes specific to *P. vulgaris*. As in other examples shown here, the availability of a fully-sequenced genome will provide a more complete picture of novel miRNAs.

Currently, microarray hybridization approaches, real time quantitative PCR (RT-qPCR) analyses and high-throughput sequencing technologies are widely used microRNA profiling methods. MiRNA regulatory functions in different organs, different stress conditions, and different developmental stages are still unknown. MiRNA profiling studies are important first approximations to analyze miRNA functions according to their different expression patterns. In this work miRNA expression was analyzed in order to find important differences in miRNA family expression levels within common bean organs. Biological replicates are essential to determine if differences observed are caused by conditions and not just by experimental variations. Because of this, cluster analysis was employed. The DESeq package allows users to work without replicates with the caveat that the test will lose strength. It assumes that most transcripts will have approximately the same levels within replicates under the different conditions, and that the estimated variance should not change too much.

Experiments designed to explore miRNA and mRNA expression are subjected to many different technical and biological biases. Nevertheless, differential processing of stem-loop precursors, small RNA duplexes, and, in general, miRNA biochemical properties challenge expression analysis methods usually used to analyze mRNAs. The majority of conserved miRNA families identified were expressed in all organs at different levels. Conserved miRNA families seem to be transcribed in almost all plant organs. Which of those conserved miRNAs have an essential role in legumes for certain developmental stages or stress responses is not completely understood. Perhaps less conserved miRNAs families that are highly abundant in legumes such as miR1510, miR1511, miR1514, miR1515, miR2118 and miR2199, play essential roles in characteristic processes of legumes such as nodulation, as altered expression of miR482 and miR1515 has been proven to increase soybean nodulation [58].

The present study contributes, together with previous common bean miRNA studies, to characterizing the *P. vulgaris *miRNA population. It represents a unique analysis of *P. vulgaris *miRNAs performed with high-throughput next-generation DNA sequencing (NGS) technologies. Shortly, full genome sequence and transcriptome datasets from different *P. vulgaris *cultivars will be available. The analysis of novel common bean genome sequence information will benefit from the tools presented here to expand important small RNA research needed in this critical worldwide crop.

## Conclusions

109 miRNAs belonging to 29 conserved families in *Phaseolus vulgaris *were identified using high-throughput sequencing. In addition, twenty six miRNA isoforms were characterized. MiRNA variant analysis identified four highly abundant miRNA families. Eleven new stem-loop precursors belonging to eight conserved miRNA families were determined. Thirty nine miRNAs identified can be explained by processing of characterized common bean precursors. In addition, twenty nine novel miRNA candidates were predicted based on small RNA reads and precursor prediction. Evidence for miRNA* sequences for four of these precursors was found. Target prediction analysis identified 157 new *Phaseolus *ESTs as established miRNA target genes in plants. Candidate targets for miRNA families derived from *Phaseolus *ESTs were proposed. The common bean miRNA families identified were differentially expressed in leaves, roots, seedlings and flowers. This work provides an important global view of conserved and novel *Phaseolus vulgaris *miRNAs, their precursors and their targets.

## Methods

### Plant material and growth conditions

*Phaseolus vulgaris *L. cv. Negro Jamapa and cv. Pinto Villa were used in this study. Common bean (cv. Negro Jamapa) seeds were surface sterilized by an initial treatment with 100% ethanol for 1 min, rinsed with sterile water, and treated with 20% sodium hypochlorite for 5 min. Sterilized seeds were transferred to sterile trays containing wet paper towels. Trays were covered with foil and held at 28°C for 72 hours until seed germination. Sprouts then were transferred to small plastic pots containing vermiculite watered with B&D solution supplemented with 8 mM KNO_3 _[59]. Incubation was performed in a chamber with 16 h of light and 8 h of dark at 28°C. Plants were watered every third day until organ collection. Roots (15 d old) and flower buds (35-40 d old) were collected in liquid N_2 _and stored at - 80°C.

*P. vulgaris *cv. Pinto Villa seeds were surface-sterilized in 50% (v/v) sodium hypochlorite and 0.5% (v/v) Tween-20 for 3 min, and rinsed with distilled water for 10 min. Seeds were transferred to trays containing wet paper towels. Whole seedlings 1-4 days old were collected in liquid N_2 _and stored at -80°C. For leaf collection, 4-day-old seedlings were transferred from trays to plastic pots containing 40% vermiculite, 30% Metro-Mix soil and 30% Agrolite. Once the first trifolium appeared, plants were kept well-watered. A pool of leaves from 10 and 20 days old was harvested for RNA purification.

### RNA isolation and small RNA library sequencing

Total RNA was isolated from frozen roots, seedlings, flower buds and leaves using the Trizol reagent according to manufacturer's instructions (Invitrogen, Carlsbad, CA). The RNA Integrity Number (RIN) was larger than seven and the 28S/18S ratio was larger than 1.6 for all sample organs. Ten micrograms of each sample (roots, flower buds, seedlings, and leaves) were prepared for Deep Sequencing following Illumina's Small RNA alternative sample preparation protocol v1.5. Small RNA fragments ranging from 18-30 nt were selected for the construction of the small RNA libraries. Complementary DNA libraries were separately Single Read-sequenced using the Genome Analyzer IIx (GAIIx)(36 bp) and the Illumina Cluster Station (Illumina Inc, USA) at the Instituto de Biotecnología (Universidad Nacional Autónoma de México).

### Small RNA sequencing analysis

Raw reads from the *Illumina Pipeline 1.4 *for the four small RNA libraries were cleaned of sequence adapters, low quality tags and small sequences (< 16 nt long). Quality analysis per cycle was performed for each library. Later, sequences were converted to FASTA format grouped in unique sequence tags with their respective frequencies. The R/Bioconductor software package ShortRead (version 1.10.4) was used for RNA sequence length distribution analysis [60]. Mature miRNA sequences from miRBase (release 16) were removed from the RNA families database (Rfam 10.0). Libraries were aligned against miRNA cleaned Rfam database using BLASTN. Exact matches identified with a Perl script for parsing blast results were then removed from libraries.

### Identification of conserved, isoform, and novel miRNAs

Mature and mature-star miRNA sequences from plants were extracted from miRBase (release 16). Sequences were grouped into unique sequences with a reference miRNA identifier. Small RNA libraries cleaned with the Rfam database were aligned against the unique mature and mature-star miRNA sequences using BLASTN and SSAHA2 (version 2.5.3). Alignment results were processed to obtain small RNA sequences that corresponded exactly in size and nucleotide composition to reported plant miRNA sequences. MiRNAs that were detected in just one library or that totalled fewer than 15 absolute appearances in all libraries were separated. Correlation coefficient analysis was done with the number of loci reported in miRBase (v.16) for *Medicago truncatula *and *Glycine max*, and the number of possible loci for *P. vulgaris *based on mature miRNA differences. With the purpose of identifying miRNA isoforms, sequences left from Rfam and miRBase filters that presented a total frequency higher than 10 in all libraries were BLASTN aligned against miRBase (mature and mature-star plant miRNA sequences) allowing at most two mismatches and/or two different nucleotides in length. The total number of variants found for each library was subjected to an abundance filter which consisted of choosing variants that had a total number of reads 50% greater than the total reads of their reference miRNA previously reported. If no reference miRNA for a variant was previously detected in all libraries, the variant with the highest frequency was considered. Isoforms detected for conserved miRNAs that belong to a miRNA family were analyzed to discard the possibility of being simply another miRNA of the same family. MiRNA isoforms were classified as length variants, non-conserved miRNA variants or conserved miRNA variants. Identification of novel miRNAs was performed with sequencing reads that remained after known miRNAs and miRNA isoforms were removed. EST and genomic sequences available from NCBI and PlantGDB were used to search for potential miRNAs using the miRDeep software [37]. Novel miRNA candidates were further aligned against nucleotide and protein NCBI databases to discard possible RNA degradation products.

### Identification of stem-loop precursors and targets

To identify stem-loop precursors, *P. vulgaris *ESTs and GSSs http://www.ncbi.nlm.nih.gov/projects/dbEST/; http://www.ncbi.nlm.nih.gov/projects/dbGSS/from NCBI were aligned against mature miRNAs, mature-star sequences and miRNA isoforms (Table 2, Table 3, Table 5 and Additional file 1) [61]. Sequence candidates with 100% coverage and identity were tested with the secondary structure program *mfold *for pre-miRNA structures [36]. Only the lowest energy structures generated for each sequence candidate were analyzed. Target prediction analysis was performed for all ESTs of the genus *Phaseolus *from NCBI. EST sequences of the genus *Phaseolus *were confirmed to be targets of mature miRNAs, mature-star sequences and miRNA isoforms (Table 2, Table 3, and Table 5) using the RNAhybrid program (version 2.1) as described by Alves et al. (2009). Potential EST targets were then aligned against plant sequences of the UniProt Knowledgebase (UniProtKB) (release 2011_01) using BLASTX for annotation. Identification analysis for common bean miR390 non-coding transcript target TAS3 was done separately. The AtTAS3 (locus: AT3G17185.1) sequence from the Arabidopsis Information Resource (TAIR) was used for BLASTN alignment against *Phaseolus *ESTs. Later, miR390 conserved binding sites were analyzed on EST candidates. Only novel mature and mature-star miRNA sequences detected in the libraries were used for target prediction analysis.

### Organ-specific expression analysis

As first step for organ-specific expression analysis, frequencies of miRNAs of the same miRNA family were added. MiRNAs considered for this analysis were the conserved miRNAs (Table 2), the five miRNAs from Additional file 1 that aligned with common bean stem-loop precursors, and the new four miRNA families proposed from the miRNA isoform study. Mature-star sequences were not considered. To test for miRNA differential expression the open-source R/Bioconductor software package DESeq was used [49]. The effective library size for all small RNA library data was estimated with the *estimateSizefactors *function. This function is used to normalize frequencies of transcripts in relation to library sizes. Then, *estimateVarianceFunctions *was used to predict the variance from the mean for each organ sample. Within these functions, the *method = "blind" *parameter for comparisons without replicates was employed. Eventually the count data was transformed with the *getVarianceStabilizedData *function such that its variance becomes independent of the mean producing a homoscedastic version of the data. Heatmap was performed using the tool heatmap.2 from the gplopts package of open source R software. Hierarchical cluster analysis was performed with the *hclust *function from the stats package.

## Competing interests

The authors declare that they have no competing interests.

## Authors' contributions

JL and PP conceived of and coordinated the study. PP wrote the manuscript. PP, MS and LP carried out the bioinformatic analyses. GE and JL carried out plant growth, RNA extraction and preparation, and helped drafting the manuscript. JL, AA and FE participated in its design, helped to draft the manuscript and provided intellectual suggestions. All authors read and approved the final manuscript.

## Supplementary Material

Additional file 1 **Conserved miRNAs detected only in one library and/or with fewer than 15 reads**. MiRNAs in common bean identified in leaves (LL), roots (RL), seedlings (SL) and flowers (FL) detected in just one library and/or with fewer than 15 total reads. Click here for file

Additional file 2 **Stem-loop miRNA precursors in common bean**. EST fragments (new conserved pre-miRNAs) and fragments of stem-loop sequences reported in miRBase (v. 16) for *P. vulgaris *where aligned against miRNA sequences (blue sequences). Count data number represents the total number of reads in all four libraries. Count data number represents the total number of reads in all four libraries. Click here for file

Additional file 3 **Novel miRNAs detected in *Phaseolus vulgaris*. **Novel miRNAs in common bean identified in leaves (LL), roots (RL), seedlings (SL) and flowers (FL) using miRDeep. Click here for file

Additional file 4 **Predicted conserved targets in *Phaseolus*. **Predicted conserved miRNA targets based on ESTs of the genus *Phaseolus*. GenBank accession numbers are used for EST identification. Calculated MFEs (kcal/mol) using RNAhybrid are shown. MiRNA families 156 and 157 share the same predicted conserved targets. MicroRNA sequences in 3'-5' sense were used to represent miRNA:target pairing. Crosses (x) and asterisks (*) denote one-nucleotide wobbles and mismatches, respectively. G:U base pairing is not considered a mismatch (|) Click here for file

Additional file 5 **Predicted Putative targets in *Phaseolus*. **Predicted putative miRNA targets based on ESTs of the genus *Phaseolus*. GenBank accession numbers are used for EST identification. Calculated MFEs (kcal/mol) using RNAhybrid are shown. MicroRNA sequences in 3'-5' sense were used to represent miRNA:target pairing. Crosses (x) and asterisks (*) denote one-nucleotide wobbles and mismatches, respectively. G:U base pairing is not considered a mismatch (|) Click here for file

Additional file 6 **Putative targets for novel miRNAs identified in *Phaseolus*. **Predicted putative targets for novel miRNAs in common bean based on EST of the genus *Phaseolus*. MicroRNA sequences in 3'-5' sense were used to represent miRNA:target pairing. Crosses (x) and asterisks (*) denote one-nucleotide wobbles and mismatches, respectively. G:U base pairing is not considered a mismatch (|) Click here for file
