# Supplementary material for: Identification and characterization of microRNAs in Phaseolus vulgaris by high-throughput sequencing
Source: BMC Genomics. 2012 Mar 6;13:83. doi: 10.1186/1471-2164-13-83 (PMC3359237; doi:10.1186/1471-2164-13-83)
Supplement: Additional file 2 — Stem-loop miRNA precursors in common bean. EST fragments (new conserved pre-miRNAs) and fragments of stem-loop sequences reported in miRBase (v. 16) for P. vulgaris where aligned against miRNA sequences (blue sequences). Count data number represents the total number of reads in all four libraries. Count data number represents the total number of reads in all four libraries. [file 1471-2164-13-83-S2.PDF]

EST (gi)	Stem-loop precursor (EST)	MiRNA Reference	Count
171606821	UUGUGCUCAAGGAAUGUAUAGUGUUGUCGGACCAGGCUUCAUUCCCCCCAAUUAUAUGCUUCCAGAAUAAUUU		
	UCGGACCAGGCUUCAUUCC	vvi-miR166a	846
	UCGGACCAGGCUUCAUUCCCC	ath-miR166a	94253
	UCGGACCAGGCUUCAUUCCC	zma-miR166h	1891
	UCGGACCAGGCUUCAUUCCCCC	ctr-miR166	40
171665565	AGUUGGGAUUAGAAGUUCGCAAAGGAAAAAGUGAAGCUGCCAGCAUGAUCUAGCUUUGGUUAGAGAGAGCAGA		
	UGAAGCUGCCAGCAUGAUCUA	ath-miR167a	6885
166970460	UCGUGCACCUGCAGCAGUUGAAGCUGCCAGCAUGAUCUGAGUUUACCUUCUAUAAUGGUAAAAACAGAUCAUG		
	UGAAGCUGCCAGCAUGAUCUGA	ccl-miR167a	13434
	UGAAGCUGCCAGCAUGAUCUG	osa-miR167d	41647
134189909	UGACUUAGUCUCAUAUGGAGCCCAUGAAUUGCUUGACAGAAGAGAGAGAGCACGGUUAACAUCUUGUGCACCA		
	UGACAGAAGAGAGAGAGCAC	ahy-miR156a	120
	UUGACAGAAGAGAGAGAGCAC	ahy-miR156c	3926
134184485	GAAAGCCAAGAAUCACUUCUAGAUGACAGAAGAGAGAGAGCACACCCUCCUGAUAAUUAUAUCAAAACAUCUC		
	UGACAGAAGAGAGAGAGCAC	ahy-miR156a	120
	UGACAGAAGAGAGAGAGCACA	osa-miR156k	2
134220273	GAUUGUGAUGAGAUAUCUCAUCUCAUGUUGACAGAAGAGAGAGAGCACAACCCGAGAAUGGUUAAAGGAGUUU		
	UUGACAGAAGAGAGAGAGCACA	gma-miR156f	2
	UGACAGAAGAGAGAGAGCACA	osa-miR156k	2
	UGACAGAAGAGAGAGAGCAC	ahy-miR156a	120
	UUGACAGAAGAGAGAGAGCAC	ahy-miR156c	3926
171642812	UACUCGAUUCUGGGCUUUGUGAUUGUUGACAGAAGAUAGAGAGCACAACCUGAGUCAAAGGAUCCAGGUUUUG		
	UUGACAGAAGAUAGAGAGCAC	ath-miR157a	15110
	UGACAGAAGAUAGAGAGCAC	ath-miR157d	506
134199621	UGUUGGUAUUUGGUGUGAAUCUACUUUGUGUUCUCAGGUCACCCCUUUGAGCCAACCUGUUGACAUGCUAAAC		
	UGUGUUCUCAGGUCACCCCU	ahy-miR398	375
	UGUGUUCUCAGGUCACCCCUU	ath-miR398a	3
171661309	AGAGAUAUCUGUUGUGGCUACGCUCAUGCACUGCCUCUUCCCUGGCUCUCUCUCUUUCUUGUUCCCUCUUCUU		
	AUGCACUGCCUCUUCCCUGG	bdi-miR408	97
	UGCACUGCCUCUUCCCUGGCU	ppt-miR408b	60
	AUGCACUGCCUCUUCCCUGGC	ath-miR408	15018
171565081	ACUGUGCGGUCUCUAAUUCGCUUGGUGCAGGUCGGGAACCUGUUUUUGCGCGAAAUGGUGGAGUGGUUGCCGG		
	UCGCUUGGUGCAGGUCGGGAA	ath-miR168a	2021
	CGGCGAAUUGGAUCCCGCCUUGCAUCAACUGAAU		
	CCCGCCUUGCAUCAACUGAAU	aly-miR168a*	113
312044525	AGGAAAACCAACCAUGUAGUUUUAAUUGAGCCGCGUCAAUAUCUCAUCUUGCACUUCUUUCCAUCUCCAACCC		
	UUGAGCCGCGUCAAUAUCUCA	pvu-isomiR171c	368

MiRBase (Acc.)	Stem-loop precursor (EST)	MIRNA Reference	Count
MI0010705	UAUUGUGUGACCCUCUUUCUCUGAGCUUGGACUGAAGGGAGCUCCUUCUUUCUGUUGCCUCCAUUUGUAUUCA		
	UUGGACUGAAGGGAGCUCC	pta-miR319	3257
	UUGGACUGAAGGGAGCUCCU	ptc-miR319e	125
	UUGGACUGAAGGGAGCUCCUU	ath-miR319c	7
	CUUGGACUGAAGGGAGCUCC	ppt-miR319a	1743
MI0010706	CUUCCAAAAGUCAGUGGCAAUAACUUGCCAAAGGAGAGUUGCCCUGUGGCUGCUUUAGCUUAAUCACAUACAG		
	UGCCAAAGGAGAGUUGCCCUG	ath-miR399b	180
MI0010700	GGAUUGAGCUGCUUAGCUAUGGAUCCCACAGUUCUACCCAUCAGCAUGUUUUGUGGUAGUCUUGUGGCUUCCA		
	AGCUGCUUAGCUAUGGAUCCC	gma-miR159d	668
	CUUCCAUAUCUGGGGAGCU	pvu-isomiR159a	456
	UAUCUGGGGAGCUUCAUCUGCCUCUAUAGUAUCAUCCUUCUUUGGAUUGAAGGGAGCUCUAC		
	UUUGGAUUGAAGGGAGCUCUA	ath-miR159a	1784126

MI0010703	UGGAUUUCUACUCUUUGAUUGGAGAAAAAAAGUGGAUUUCUAUUUUUUUCAAUCAAAGGGAGUUGUAGGGGAA		
	UCAAAGGGAGUUGUAGGGGAA	gma-miR2119	1581
MI0010702	GGGAUCCGGAGAUUGGAGCAAUCAGAAUUUGUGGGAAUGGGCUGAUUGGGAAGCAAUGUGUGCUGGUGCAAUG		
	AGAAUUUGUGGGAAUGGGCUGA	gma-miR482a-5p	19
	GGAAUGGGCUGAUUGGGAAGCA	pvu-miR482*	1641
	GGAAUGGGCUGAUUGGGAAGC	pvu-isomiR482*	1207244
	CAUUUAAUUUCUUCCCAAUUCCGCCCAUUCCUAUGAU		
	UCUUCCCAAUUCCGCCCAUUCC	pvu-miR482	350
	UCUUCCCAAUUCCGCCCAUUCCUA	gma-miR482a-3p	2
MI0010704	GGAAUGUUGUCUGGCUCGAGGUCAUGGAGGAGGAGAGUUCUCAGAUAAACUCUUUCACCCAAAGUUUCCAAUG		
	GGAAUGUUGUCUGGCUCGAGG	gma-miR166a-5p	70860
	GAAAUUUACCCUCUAACACCAAAAUGAUUCUCGGACCAGGCUUCAUUCCCCCCACC		
	UCGGACCAGGCUUCAUUCC	vvi-miR166a	846
	UCGGACCAGGCUUCAUUCCCC	ath-miR166a	94253
	UCGGACCAGGCUUCAUUCCC	zma-miR166h	1891
	UCGGACCAGGCUUCAUUCCCCC	ctr-miR166	40
MI0010701	UUUUGAAUGGAAACAGGGCUUUCCUGUUUUCAUUUUGAAAAUAGGCAUUGCAUUACAUUUCGAUGCGUCUUCC		
	UUCAUUUUGAAAAUAGGCAUUG	pvu-miR1514a	7262
MI0010699	AUGAAUAUUUCUCACUCUGUUUUGCCGAUUCCACCCAUUCCUAUGAUUUCCUUUGGUUCCUUUCUUUCCACUC		
	UUGCCGAUUCCACCCAUUCCUA	pvu-miR2118	33016
